# Clinicopathologic Significance of *VHL* Gene Alteration in Clear-Cell Renal Cell Carcinoma: An Updated Meta-Analysis and Review

**DOI:** 10.3390/ijms19092529

**Published:** 2018-08-26

**Authors:** Hyeong Su Kim, Jung Han Kim, Hyun Joo Jang, Boram Han, Dae Young Zang

**Affiliations:** Department of Internal Medicine, Hallym University Medical Center, Hallym University College of Medicine, Seoul 07441, Korea; nep2n@hallym.or.kr (H.S.K.); jhj1229@hallym.or.kr (H.J.J.); borbor@hallym.or.kr (B.H.); fhdzang@hallym.or.kr (D.Y.Z.)

**Keywords:** renal cell carcinoma, *von Hippel-Lindau*, mutations, methylation, meta-analysis, review

## Abstract

The *von Hippel-Lindau* (*VHL*) gene is inactivated frequently in sporadic clear-cell renal cell carcinomas (ccRCCs) by genetic alteration (mutation, loss of heterozygosity, or promoter hypermethylation). However, the pathological or prognostic significance of *VHL* gene alteration has not been well defined. We conducted this meta-analysis to evaluate the association between *VHL* alteration and clinopathologic findings in ccRCCs. We performed a systematic computerized search of online databases, including PubMed, EMBASE, Web of Science, and Google Scholar (up to July 2018). From ten studies, 1,082 patients were included in the pooled analyses of odds ratios (ORs) with 95% confidence intervals (CIs) for pathological features (nuclear grade and disease stage) or hazard ratios (HRs) with 95% CIs for overall survival (OS). *VHL* alteration was not significantly associated with nuclear grade (OR = 0.79, 95% CI: 0.59–1.06, *p* = 0.12) or disease stage (OR = 1.07, 95% CI: 0.79–1.46, *p* = 0.65). There was also no significant correlation between *VHL* alteration and OS (HR = 0.75, 95% CI: 0.43–1.29, *p* = 0.30). When we pooled HRs for OS according to the *VHL* alteration types, the combined HRs were 0.72 (95% CI: 0.47–1.11, *p* = 0.14) for *VHL* mutations and 1.32 (95% CI: 0.70–2.47, *p* = 0.39) for methylation. In conclusion, this meta-analysis indicates that *VHL* gene alteration is not significantly associated with the pathological features and survival in patients with ccRCC.

## 1. Introduction

Renal cell carcinoma (RCC) is the second common malignancy after bladder cancer in the urinary system, accounting for approximately 63,000 and 5000 new cases diagnosed each year in the United States and South Korea, respectively [1,2]. RCCs comprise a group of heterogeneous tumors with distinct molecular alterations. Clear-cell RCC (ccRCC) is the most common histotype representing 75% of cases, followed by papillary RCCs (12%), chromophobic RCC (4%), oncocytomas (4%), and rare subtypes [3]. Complete surgical resection is considered to be a curative treatment option for RCCs at the early or locally advanced stage. However, about 30% of patients who received curative resection experience disease recurrence [4]. Despite the recent introduction of new molecular targeted agents, the prognosis of recurrent or metastatic RCC is very poor, with 5-year survival rate of about 10% [5,6,7,8].

The *von Hippel-Lindau* (*VHL*) gene is a tumor suppressor gene that was identified in patients with VHL syndrome [9]. *VHL* gene has an important role in regulation of the hypoxia pathway via the hypoxia inducible factors (HIFs) in sporadic RCCs [10,11]. Functional loss of *VHL* protein (pVHL), which is induced from *VHL* gene alteration, allows HIFs to act as a transcription factor of various pro-tumorigenic genes including vascular endothelial growth factor (VEGF) and subsequently leads to renal tumorigenesis and progression by inducing angiogenesis [12]. The *VHL* gene alteration events include *VHL* gene mutation, promoter hypermethylation, and loss of heterozygosity (LOH) by allele deletion with concomitant alteration of the contralateral gene. These genetic or epigenetic alterations play a major role in the deregulated expression of *VHL* gene. Somatic *VHL* mutations are identified in 30–60% of ccRCCs, accounting for the vast majority of sporadic ccRCCs [13]. The majority of ccRCCs containing somatic *VHL* mutations also exhibits contralateral allele loss (LOH), consistent with a two-hit hypothesis of tumorigenesis [13,14]. Together with 3p LOH, *VHL* mutation is considered a rate-limiting event in the development of RCC [13,15]. Hypermethylation of DNA in the promoter region of *VHL* gene results in transcriptional gene silencing. *VHL* promoter methylation has been observed in 10–20% of sporadic ccRCCs [16,17].

Since Neumann et al. reported that RCCs developing in patients with VHL disease had a better prognosis than sporadic RCCs [18], a series of studies investigated the correlation of somatic *VHL* alteration with pathological characteristics or prognosis in ccRCCs [18,19,20,21,22,23,24,25,26,27,28,29,30,31]. Some studies found that *VHL* alteration correlated with favorable pathological features or prognosis [22,28,29,30]. However, Brauch et al. observed an association of *VHL* alteration with advanced tumor stage [19]. Salinas-Sánchez et al. reported that *VHL* methylation was associated with worse survival [31]. Other studies failed to demonstrate the pathological or prognostic significance of *VHL* alteration in patients with ccRCC [19,20,21,23,24,25,26,27].

However, most individual studies had a small sample size and the statistical power was limited. Therefore, we conducted this meta-analysis to gain a better insight into the clinicopathologic significance of *VHL* gene alteration in patients with ccRCC.

## 2. Results

### 2.1. Results of Search

**Figure 1:**
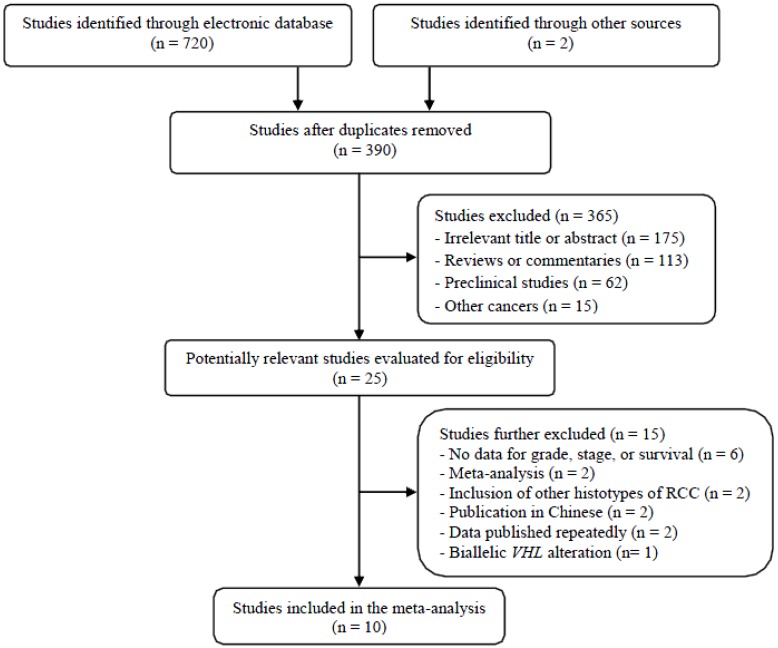
Flow diagram of search process.

Figure 1 shows the flow diagram of the search process. A total of 722 potentially relevant articles were initially retrieved, but 697 of them were excluded after careful screening of the titles and abstracts. Of the remaining 25 potentially eligible studies, 15 were further excluded by the inclusion criteria. Eventually, the remaining 10 studies with 1082 ccRCC patients were included in the meta-analysis [22,23,24,25,26,27,28,29,30,31].

### 2.2. Characteristics of the Included Studies

Table 1 summarizes the main characteristics and clinicopathologic data of the 11 included studies. Most studies were performed retrospectively and published between 2001 and 2017. DNA-single-strand conformation polymorphism (DNA-SSCP) or polymerase chain reaction (PCR) followed by direct sequencing was used to detect *VHL* mutations, and methylation-specific PCR was used to assess *VHL* promoter methylation status. The incidence of *VHL* gene alteration including mutations, methylation, or LOH was various from 28.6% [24] to 88.8% [29]: mutations ranged from 26% [30] to 72.2% [28] and methylation ranged from 16% [23] to 31.3% [28].

While four studies reported *VHL* alteration as a favorable pathological or prognostic factor [22,28,29,30], one observed its association with worse survival [31]. The remaining five studies failed to demonstrate the significant pathological or prognostic value of *VHL* alteration in ccRCCs [23,24,25,26,27].

### 2.3. Impact of VHL Gene Alteration on Pathological Features

**Figure 2:**
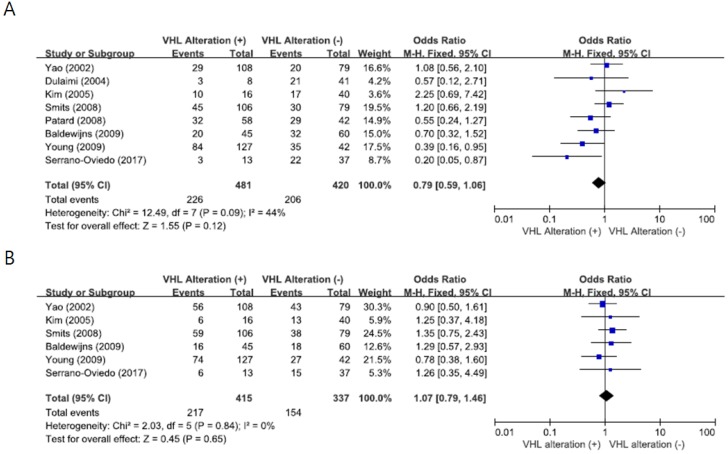
Forest plots for the association between *VHL* alteration and pathological features. (**A**) Nuclear grade. (**B**) Disease stage.

From eight studies [22,23,24,25,26,27,28,30], 393 patients were included in the meta-analysis of ORs with 95% CIs for nuclear grade (NG). There was no substantial heterogeneity across the studies (*X*^2^ = 12.49, *p* = 0.09, *I^2^* = 44%) and the fixed-effect model was used. There was no significant correlation between *VHL* alteration and NG (III-IV) (OR = 0.79, 95% CI: 0.59-1.06, *p* = 0.12) (Figure 2A).

From six studies [22,24,25,27,28,30], 415 patients were analyzed for the impact of *VHL* alteration on disease stage. The fixed-effect model was selected because there was no heterogeneity across the studies (*X^2^* = 2.03, *p* = 0.84, *I*^2^ = 0%). There was also no significant association between *VHL* alteration and disease stage (III-IV) (OR = 1.07, 95% CI: 0.79-1.46, *p* = 0.65) (Figure 2B).

### 2.4. Impact of VHL Variations on Overall Survival

**Figure 3:**
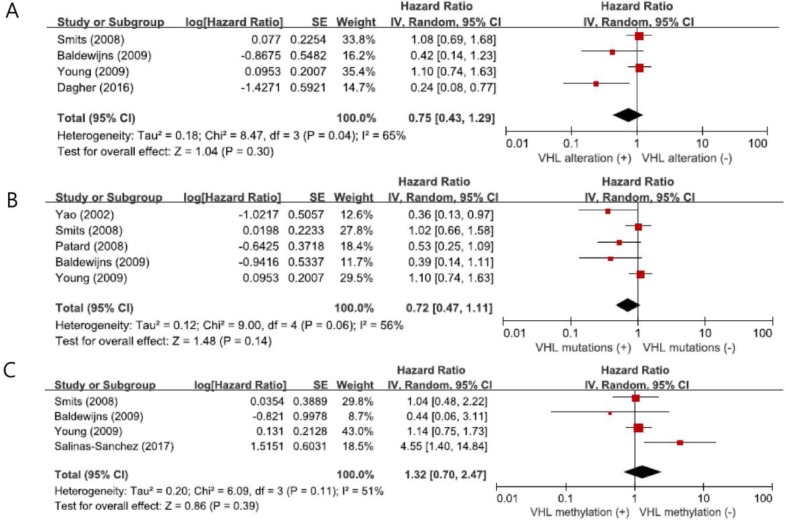
Forest plots for the association of *VHL* variations with overall survival. (**A**) *VHL* alteration. (**B**) *VHL* mutations. (**C**) *VHL* methylation.

From four studies [25,27,28,29], a total of 593 patients with *VHL* alteration (mutation, LOH, or methylation) were included in the meta-analysis of HRs for OS. There was a significant heterogeneity among the studies (*X*^2^ = 8.47, *p =* 0.04, *I*^2^ = 65%) and the random-effects model was selected. *VHL* alteration showed no significant impact on OS (HR = 0.75, 95% CI: 0.43-1.29, *p* = 0.30) (Figure 3A). 

When we pooled HRs for OS according to the *VHL* alteration type (mutations or methylation), the combined HRs were 0.72 (95% CI: 0.47-1.11, *p* = 0.14, *I*^2^ = 56%, random-effects model) for *VHL* mutations (Figure 3B) and 1.32 (95% CI: 0.70-2.47, *p* = 0.39, *I*^2^ = 51%, random-effects model) for methylation (Figure 3C), indicating no significant prognostic impact in ccRCCs.

### 2.5. Publication Bias

**Figure 4:**
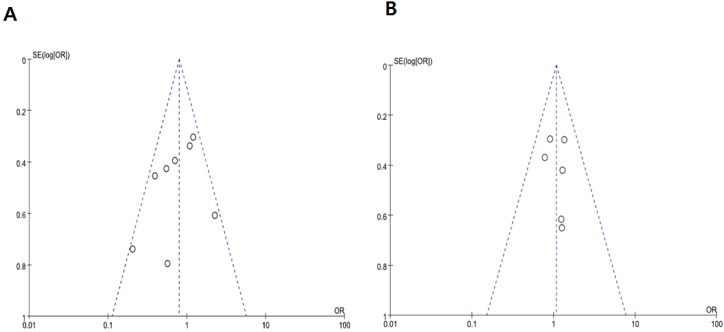
Funnel plots for publication bias. (**A**) Nuclear grade. (**B**) Disease stage.

We did not perform publication bias tests for OS because a limited number of studies were included. Visual inspection of the funnel plots for NG and stage showed symmetry, suggesting there was no substantial publication bias (Figure 4A,B). Egger’s tests also indicated the absence of significant publication biases (*p* = 0.357 for NG and *p* = 0.658 for disease stage).

## 3. Discussion

Although *VHL* gene alteration accounts for the vast majority of sporadic ccRCCs and provides plausible therapeutic target for anti-angiogenic agents, its clinicopathologic significance is still controversial with conflicting results among studies. In the current meta-analysis, we evaluated the pathological and prognostic value of *VHL* alteration in patients only with ccRCC. Our results failed to demonstrate the significant association of *VHL* alteration with pathological features and prognosis in ccRCCs.

From an improved understanding of the molecular biology of RCCs during the last decade, new molecular targeted agents have emerged for patients with advanced RCC [5,6,7,8,17]. *VHL* gene encodes a multifunctional protein (pVHL) which has an important role in regulation of the hypoxia pathway via the HIFs in sporadic RCCs [11]. With functional loss of pVHL arising from the genetic or epigenetic changes of *VHL* (*VHL* alteration), HIFs can act as a transcription factor of various pro-tumorigenic genes including VEGF [12]. Recently introduced targeted agents (bevacizumab, sunitinib, sorafenib, regorafenib, or pazopanib) for RCCs have anti-angiogenic effects to modulate this VHL-HIF pathway [5,6,7,8,32,33,34]. Therefore, it is logical to assume that *VHL* alteration may have important implications for disease prognosis.

The clinicopathologic impacts of somatic *VHL* mutations or promoter methylation have been studied in a variety of case series with RCCs [18,19,20,21,22,23,24,25,26,27,28,29,30,31]. Many studies have failed to observe the pathological or prognostic significance of *VHL* alteration in patients with ccRCC [19,20,21,23,24,25,26,27]. However, some investigators published the results suggesting a significant association of *VHL*-altering events with the pathological or survival outcomes [19,22,29,31]. First, Brauch et al. analyzed *VHL* alteration in a retrospective cohort of 227 sporadic RCC [19]. *VHL* mutations or promoter hypermethylation were identified in 45% of ccRCC and the rate of LOH was 93% of the cases. The presence of *VHL* alteration significantly correlated with a standard prognostic factor, pT3 tumor stage (*p* = 0.009). Recently, Salinas-Sánchez et al. conducted a prospective, longitudinal cohort study of 50 patients diagnosed with ccRCC and analyzed *VHL* mutations and hypermethylation as well as VHL, HIF1-α, VEGF, ERK1/2 and ERK5 protein expression [31]. *VHL* mutations and methylation were identified in 26.5% and 21.7% of cases, respectively. Disease-specific survival (DSS) was greater in patients without *VHL* methylation (*p* = 0.012), with > 10% of HIF1-α expression (*p* = 0.037), or with ERK5 underexpression (*p* = 0.018). Contrary to the findings above, however, *VHL* alteration has shown favorable survival outcomes in other studies [22,29]. Yao et al. analyzed 187 Japanese patients with sporadic ccRCC for *VHL* alteration events (mutations and hypermethylation) [22]. *VHL* alteration was detected in 108 tumor samples: intragenic mutations in 98 patients (52%) and hypermethylation in 10 (5.3%). *VHL* mutations were associated with better DSS in patients with stage 1-III disease, but not in those with stage IV disease, suggesting that *VHL* mutation may contribute most before the disease proceed to advanced or metastatic stage. Recently, Dagher et al. performed a retrospective cohort study of 98 patients with ccRCC who underwent radical surgery between 2001 and 2005 [29]. At least one or more *VHL* abnormalities were detected in 87 cases (88.8%): mutations in in 68 patients (69.4%), LOH in 71 (72.4%), and hypermethylation in 13 (13.3%). Patients with tumors of wild-type *VHL* were associated with nodal involvement (*p* = 0.019) and showed worse DSS compared with those with ccRCC carrying one or two *VHL* inactivating events (33 months vs. 107 months, *p* = 0.016).

There are two meta-analyses in the literature that investigated the pathological or prognostic impact of *VHL* alteration in RCC [35,36]. Our previous study evaluated the association of *VHL* alteration and clinical outcomes in patients with ccRCC and found that *VHL* alteration had no predictive or prognostic values [35]. However, we included only 3 studies in the meta-analysis and did not evaluate the pathologic impact of *VHL* alteration. Yang et al. investigated pathologic significance of *VHL* promoter hypermethylation with 13 studies and found that *VHL* hypermethylation was not correlated with specific clinicopathologic characteristics [36]. However, they included studies with other histotypes of RCC which rarely carry *VHL* alteration and did not evaluate the prognostic significance of *VHL* methylation. Ultimately, it is not clear that *VHL* gene alteration has specific pathological or prognostic value in sporadic ccRCCs. Most studies to date have been limited mainly by the retrospective design with a small number of patients. To give more statistical power, we conducted this comprehensive meta-analysis comprising an extended number of studies. Because the vast majority of *VHL* alteration events usually occur in ccRCCs, we excluded studies with patients diagnosed with other histotypes of RCC [20,37]. We systematically investigated the pathological and survival data of 1,082 patients with ccRCC from the ten studies [22,23,24,25,26,27,28,29,30,31]. Our results revealed that *VHL* alteration was not significantly associated with NG (OR = 0.79, 95% CI: 0.59–1.06, *p* = 0.12) and disease stage (OR = 1.07, 95% CI: 0.79–1.46, *p* = 0.65). In addition, there was no significant association between *VHL* alteration and prognosis of ccRCCs (HR = 0.75, 95% CI: 0.43–1.29, *p* = 0.30). These results indicate that *VHL* alteration has no significant pathological or prognostic impact in patients with ccRCC.

As mentioned above, *VHL* gene alteration is a broad concept of genetic abnormality which includes mutations, promoter hypermethylation, and LOH. Thus, we further performed the subgroup analyses to determine the difference of the polled HR for two major *VHL* alteration events (mutation and hypermethylation). However, the results showed that both mutation (HR = 0.72, 95% CI: 0.47–1.11, *p* = 0.14) and hypermethylation (HR = 1.32, 95% CI: 0.70–2.47, *p* = 0.39) were not significantly correlated with prognosis of ccRCCs. Recently, Lessi et al. reported an interesting result regarding *VHL* gene variation and prognosis in early-stage ccRCCs [38]. Tumors carrying biallelic alterations (mutation in homozygosis or presence at the same time of LOH and mutation, LOH and methylation, or methylation and mutation) had a shorter DSS compared with those with no *VHL* alteration. However, as the study had too small number of cases with biallelic alterations (only 15) to reach statistical significance, large sample size studies are warranted to exploit this issue.

There are several mutation types such as silent, nonsense, missense, and frameshift mutation. Loss of function (LOF) mutations can be defined as events which alter *VHL* transcriptional read through such as nonsense or frameshift mutations predicted to interfere with protein stability and missense mutation which alter *VHL* start codon [38]. Several studies reported that LOF mutations rather than other types of *VHL* alteration showed a meaningful correlation with worse survival [24,39]. However, these findings are premature to accept due to the small number of cases that contains LOF mutations. There are also other studies which failed to observe the significant correlation between LOF mutations and survival [25,27]. Interestingly, LOF mutations acted as a good predictive marker for VEGF-targeted therapy in patients with RCC [34,40]. Choueiri et al. investigated the *VHL* gene status and response to VEGF-targeted agents in patients with metastatic ccRCC [34]. In patients with tumors containing *VHL* mutations or methylation, the response rate was 37% compared with 31% in the wild-type group (*p* = 0.34). However, patients with LOF mutations obtained a significantly higher response rate than those with *VHL*-wild-type tumor (52% vs. 31%, *p* = 0.04). In this meta-analysis, we could not evaluate the impact of LOF mutations as a prognostic marker because *VHL* mutations were not classified into the subgroups in most studies.

This study has some inherent limitations that need to be discussed. First, this meta-analysis included a small number of studies. Second, almost all studies were retrospectively performed and therefore might carry the biases of the retrospective design. Third, patients had several potential confounders, such as ethnicities, tumor stage, and modalities of treatment. Forth, the substantial heterogeneity observed across the studies could not be completely interpreted although the random-effects model was selected for pooling HRs for OS. Finally, only articles published in English were included, which might have led to selection bias.

In conclusion, this meta-analysis indicates that *VHL* gene alteration is not significantly associated with the pathological features or survival in patients with ccRCC. However, large-scale studies are needed to reveal the predictive or prognostic role according to the mutational subtypes of *VHL* gene in patients with ccRCC.

## 4. Materials and Methods

### 4.1. Searching Strategy

This meta-analysis was conducted according to the Preferred Reporting Items for Systematic Reviews and Meta-Analyses (PRISMA) guidelines [41]. We performed a systematic computerized search of online databases, including PubMed, EMBASE, Web of Science, and Google Scholar (up to July 2018). The following searching terms were used: “kidney” or “renal” and “carcinoma” or “cancer” or “neoplasm” or “malignancy” and “*von Hippel-Lindau*” or “*VHL*” and “alteration” or “mutation” or “methylation” or “loss of heterozygosity.” All eligible studies were retrieved and their bibliographies were checked for other relevant publications. Reference lists of identified studies and reviews were also hand-searched.

### 4.2. Inclusion Criteria

Eligible studies should meet the following inclusion criteria: (i) prospective or retrospective cohort studies investigating the correlation of *VHL* alteration (mutation, LOH, or hypermethylation) with pathological features or survival in patients with histologically confirmed ccRCC; (ii) the use of tumor tissues and adequate methods to assess *VHL* alteration events; (iii) sufficient data for odds ratio (OR) with 95% confidence interval (CI) for pathological findings or hazard ratio (HR) with 95% CI for overall survival (OS); (iv) studies published only in peer-reviewed journals; and (v) articles written in English.

### 4.3. Data Extraction

Two investigators (H.S.K and H.J.J.) independently screened relevant studies and extracted the data from each eligible study. If these two researchers did not agree, the principle investigator (J.H.K.) was consulted to resolve the disagreement through discussion.

The following data were extracted from the included studies: first author, year of publication, country, number of patients, methods for *VHL* gene alteration, and pathological or survival data for ORs and HRs with their 95% CIs. When both univariate and multivariate analysis were performed to get the HR for OS, the data from multivariate analysis were extracted preferentially.

### 4.4. Statistical Analyses

Statistical values were directly extracted from the original articles or were indirectly estimated from the given data. If HRs or ORs with their 95% CIs were not presented, the Engauge Digitizer software was used to estimate them from the corresponding data and Kaplan-Meier curves, respectively. The strength of the association between *VHL* alteration and pathological features was shown as ORs and their 95% CIs.

The RevMan version 5.3 (Cochrane Collaboration, Copenhagen, Denmark) was used to combine the data. The plots show a summary estimate of the results from all studies combined. The size of each square represents the estimate from each study, reflecting the statistical “weight” of the study. Outcomes are presented as forest plots with diamonds representing the estimate of the pooled effect. The width of each diamond implies its precision. The line of no impact is number one for binary outcomes, which depicts statistical significance if not crossed by the diamond [42]. The heterogeneity across studies was estimated by the Cochran’s *Q* statistics and *I*^2^ inconsistency test. The fixed-effect model (Mantel–Haenszel method) was used for pooling homogeneous outcomes (*p* ≥ 0.1 or *I*^2^ ≤ 50%), and the random-effects model (DerSimonian–Laird method) was selected in cases with significant heterogeneity (*p* < 0.1 and *I*^2^ > 50%). Statistical significance of the pooled HR or OR was determined by the *Z*-test for overall effect. The pooled OR or HR < 1.0 implies better pathological or survival outcomes for ccRCCs with *VHL* alteration.

Publication bias was evaluated by funnel plot and quantified by the Egger’s test to assess funnel plot asymmetry [43,44]. Statistical significance was considered for a *p*-value of less than 0.05.

## Figures and Tables

**Table 1:** Summary of the 10 included studies.

First Author (Year) [Ref]	Country	Detection Methods	No. of Patients	*VHL* Alteration (Yes, No) (%)	Grade III–IV (Yes vs. No) *p*-Value OR for Grade III–IV (95% CI)	Stage III–IV (Yes vs. No) *p*-Value OR for Stage III–IV (95% CI)	HR for OS (95% CI) *p*-Value
Yao (2002) [22]	Japan	DNA-SSCP + direct sequencing	187	Mutations (yes: 98, no: 89) (52.4%) Methylation (yes: 10, no 177) (5.3%)	29/108 (26.9%) vs. 20/79 (25.3%) *p* = 0.463 1.08 (0.56–2.10)	56/108(51.8%) vs. 43/79 (54.4%) *p* = 0.463 0.9 (0.50–1.61)	Mutations: 0.36 (0.13–0.97) *p* = 0.023
Dulaimi (2004) [23]	USA	MS-PCR	50	Methylation (yes: 8, no: 42) (16%)	3/8(37.5%) vs. 21/41 (51.2%) NS 0.57 (0.12–2.71)	NA	NA
Kim (2005) [24]	Korea	DNA-SSCP + direct sequencing MS-PCR	56	Mutations or methylation (yes: 16, no: 40) (28.6%)	10/16 (62.5%) vs. 17/40 (42.5%) *p* = 0.487 2.25 (0.69–7.42)	6/16 (37.5%) vs. 13/40 (32.5%) *p* = 0.809 1.25 (0.37–4.18)	NA
Smits (2008) [25]	Netherlands	DNA-SSCP + direct sequencing MS-PCR	185	LOF mutations or methylation (yes: 106, no: 79) (57.3%) Methylation (yes: 16, no: 131) (10.9%)	45/106 (42.5%) vs. 30/79 (38%) *p* = 0.537 1.20 (0.66–2.19)	59/106 (55.7%) vs. 38/79 (48.1%) *p* = 0.681 1.35 (0.75–2.43)	1.08 (0.69–1.68) *p* = 0.461 Mutations: 1.02 (0.66–1.58) Methylation: 1.04 (0.48–2.22) *p* = 0.439
Patard (2008) [26]	France, USA	PCR + direct sequencing	100	Mutations (yes: 58, no: 42) (58%)	32/58 (55.2%) vs. 29/42 (69%) *p* = 0.16 0.55 (0.24–1.27)	NA	0.53 (0.25–1.09) *p* = 0.084
Baldewijns (2009) [27]	Belgium	PCR + direct sequencing MS-PCR	134	LOF mutations or methylation (yes: 96, no: 38) (71.6%)	20/45 (44.4%) vs. 32/60 (53.5%) *p* = 0.762 0.70 (0.32–1.52)	16/45 (35.6%) vs. 18/60 (30%) *p* = 0.212 1.29 (0.57–2.93)	0.42 (0.14–1.23) *p* = 0.423 Mutations: 0.39 (0.14–1.11) *p* = 0.078 Methylation: 0.44 (0.06–3.11) *p* = 0.411
Young (2009) [28]	UK	PCR + DNA sequencing MS-PCR	177	Mutations (yes: 127, no: 42) (75.1%) Methylation (yes: 51, no: 112) (31.3%)	84/127 (66.1%) vs. 35/42 (83.3%) 0.39 (0.16–0.95) 42/51 (82.4%) vs. 71/112 (63.4%)	74/127 (58.3%) vs. 27/42 (64.3%) 0.78 (0.38–1.60) 33/51 (64.7%) vs. 66/112 (58.9%)	1.10 (0.74–1.63) *p* = 0.63 Mutations: 0.87 (0.30–2.56) *p* = 0.80 Methylation: 1.14 (0.75–1.73) *p* = 0.54
Dagher (2016) [29]	France	PCR + sequencing reaction MS-MLPA	98	Mutations, LOH, or methylation (yes: 87, no: 11) (88.8%)	NA	NA	0.24 (0.075–0.766) *p* = 0.016
Serrano-Oviedo (2017) [30]	Spain	PCR + direct sequencing	50	Mutations (yes: 13, no: 37) (26%)	3/13 (23.1%) vs. 22/37 (59.5%) *p* = 0.036 0.20 (0.05–0.87)	6/13 (46.2%) vs. 15/37 (40.5%) NS 1.26 (0.35–4.49)	NA
Salinas-Sanchez (2017) [31]	Spain	PCR + automatic DNA sequencing MS-PCR	46	Methylation (yes: 10, no: 36) (21.7%)	NA	NA	Methylation: 4.45 (1.40–14.84) *p* = 0.012

*VHL*, *von Hippel-Lindau*; DNA-SSCP, DNA-single-strand conformation polymorphism; MS-PCR, methylation-specific polymerase chain reaction; MS-MLPA, methylation-specific multiplex ligation-dependent probe amplification; LOF, loss of function; LOH, loss of heterozygosity; OR, odds ratio; HR, hazard ratio; CI, confidence interval; OS, overall survival; NS, not significant; NA, not available.

## Abbreviations

ccRCC	clear-cell renal cell carcinoma
CI	confidence interval
DSS	disease-specific survival
HR	hazard ratio
LOF	loss of function
LOH	loss of heterozygosity
NG	nuclear grade
OR	odd ratio
OS	overall survival
PCR	polymerase chain reaction
*VHL*	*von Hippel-Lindau*
